# Supplementary material for: A UMLS-based spell checker for natural language processing in vaccine safety
Source: BMC Med Inform Decis Mak. 2007 Feb 12;7:3. doi: 10.1186/1472-6947-7-3 (PMC1805499; doi:10.1186/1472-6947-7-3)
Supplement: Additional file 13 — RAP application directory. Sets up directory for RAP (RDF application for PHP) source code [file 1472-6947-7-3-S13.gz › rap/doc/phpdoc/__filesource/fsource_default__vocabularyDC_C.php.html]

xml version="1.0" encoding="iso-8859-1"?


File Source for DC\_C.php


# Source for file DC\_C.php

Documentation is available at DC\_C.php

```
1. <?PHP
2. // ----------------------------------------------------------------------------------
3. // Dublin Core Vocabulary (Resource)
4. // ----------------------------------------------------------------------------------
5. // Version                   : 0.9
6. // Authors                   : Chris Bizer (chris@bizer.de)
7. //
8. // Description               : Wrapper, defining resources for all terms of the Dublin
9. //                               Core Vocabulary. For details about DC see: http://dublincore.org/
10. //                                Using the wrapper allows you to define all aspects of
11. //                             the vocabulary in one spot, simplifing implementation and
12. //                             maintainence.
13. //
14. // ----------------------------------------------------------------------------------
15. // <BR><BR>History:<UL>
16. // <LI>07-26-2004                 : Update for latest DC recs; changed suffixed globals to
17. //                                  array items.(auer@informatik.uni-leipzig.de)</LI>
18. // <LI>02-21-2003                 : Initial version (evan@wikitravel.org)</LI>
19. // ----------------------------------------------------------------------------------


29. class DC{
30. // DC concepts

32. function CONTRIBUTOR()
33. {
34. return  new Resource(DC_NS . 'contributor');

36. }

38. function COVERAGE()
39. {
40. return  new Resource(DC_NS . 'coverage');

42. }

44. function CREATOR()
45. {
46. return  new Resource(DC_NS . 'creator');

48. }

50. function DATE()
51. {
52. return  new Resource(DC_NS . 'date');

54. }

56. function DESCRIPTION()
57. {
58. return  new Resource(DC_NS . 'description');

60. }

62. function FORMAT()
63. {
64. return  new Resource(DC_NS . 'format');

66. }

68. function IDENTIFIER()
69. {
70. return  new Resource(DC_NS . 'identifier');

72. }

74. function LANGUAGE()
75. {
76. return  new Resource(DC_NS . 'language');

78. }

80. function PUBLISHER()
81. {
82. return  new Resource(DC_NS . 'publisher');

84. }

86. function RIGHTS()
87. {
88. return  new Resource(DC_NS . 'rights');

90. }

92. function SOURCE()
93. {
94. return  new Resource(DC_NS . 'source');

96. }

98. function SUBJECT()
99. {
100. return  new Resource(DC_NS . 'subject');

102. }

104. function TITLE()
105. {
106. return  new Resource(DC_NS . 'title');

108. }

110. function TYPE()
111. {
112. return  new Resource(DC_NS . 'type');
113. }


116. // Other Elements and Element Refinements

118. function ABSTRACT_()
119. {
120. return  new Resource(DCTERM_NS . 'abstract');

122. }

124. function ACCESS_RIGHTS()
125. {
126. return  new Resource(DCTERM_NS . 'accessRights');

128. }

130. function ALTERNATIVE()
131. {
132. return  new Resource(DCTERM_NS . 'alternative');

134. }

136. function AUDIENCE()
137. {
138. return  new Resource(DCTERM_NS . 'audience');

140. }

142. function AVAILABLE()
143. {
144. return  new Resource(DCTERM_NS . 'available');

146. }

148. function BIBLIOGRAPHIC_CITATION()
149. {
150. return  new Resource(DCTERM_NS . 'bibliographicCitation');

152. }

154. function CONFORMS_TO()
155. {
156. return  new Resource(DCTERM_NS . 'conformsTo');

158. }

160. function CREATED()
161. {
162. return  new Resource(DCTERM_NS . 'created');

164. }

166. function DATE_ACCEPTED()
167. {
168. return  new Resource(DCTERM_NS . 'dateAccepted');

170. }

172. function DATE_COPYRIGHTED()
173. {
174. return  new Resource(DCTERM_NS . 'dateCopyrighted');

176. }

178. function DATE_SUBMITTED()
179. {
180. return  new Resource(DCTERM_NS . 'dateSubmitted');

182. }

184. function EDUCATION_LEVEL()
185. {
186. return  new Resource(DCTERM_NS . 'educationLevel');

188. }

190. function EXTENT()
191. {
192. return  new Resource(DCTERM_NS . 'extent');

194. }

196. function HAS_FORMAT()
197. {
198. return  new Resource(DCTERM_NS . 'hasFormat');

200. }

202. function HAS_PART()
203. {
204. return  new Resource(DCTERM_NS . 'hasPart');

206. }

208. function HAS_VERSION()
209. {
210. return  new Resource(DCTERM_NS . 'hasVersion');

212. }

214. function IS_FORMAT_OF()
215. {
216. return  new Resource(DCTERM_NS . 'isFormatOf');

218. }

220. function IS_PART_OF()
221. {
222. return  new Resource(DCTERM_NS . 'isPartOf');

224. }

226. function IS_REFERENCED_BY()
227. {
228. return  new Resource(DCTERM_NS . 'isReferencedBy');

230. }

232. function IS_REPLACED_BY()
233. {
234. return  new Resource(DCTERM_NS . 'isReplacedBy');

236. }

238. function IS_REQUIRED_BY()
239. {
240. return  new Resource(DCTERM_NS . 'isRequiredBy');

242. }

244. function ISSUED()
245. {
246. return  new Resource(DCTERM_NS . 'issued');

248. }

250. function IS_VERSION_OF()
251. {
252. return  new Resource(DCTERM_NS . 'isVersionOf');

254. }

256. function LICENSE()
257. {
258. return  new Resource(DCTERM_NS . 'license');

260. }

262. function MEDIATOR()
263. {
264. return  new Resource(DCTERM_NS . 'mediator');

266. }

268. function MEDIUM()
269. {
270. return  new Resource(DCTERM_NS . 'medium');

272. }

274. function MODIFIED()
275. {
276. return  new Resource(DCTERM_NS . 'modified');

278. }

280. function REFERENCES()
281. {
282. return  new Resource(DCTERM_NS . 'references');

284. }

286. function REPLACES()
287. {
288. return  new Resource(DCTERM_NS . 'replaces');

290. }

292. function REQUIRES()
293. {
294. return  new Resource(DCTERM_NS . 'requires');

296. }

298. function RIGHTS_HOLDER()
299. {
300. return  new Resource(DCTERM_NS . 'rightsHolder');

302. }

304. function SPATIAL()
305. {
306. return  new Resource(DCTERM_NS . 'spatial');

308. }

310. function TABLE_OF_CONTENTS()
311. {
312. return  new Resource(DCTERM_NS . 'tableOfContents');

314. }

316. function TEMPORAL()
317. {
318. return  new Resource(DCTERM_NS . 'temporal');

320. }

322. function VALID()
323. {
324. return  new Resource(DCTERM_NS . 'valid');

326. }


329. // Encoding schemes

331. function BOX()
332. {
333. return  new Resource(DCTERM_NS . 'Box');

335. }

337. function DCMI_TYPE()
338. {
339. return  new Resource(DCTERM_NS . 'DCMIType');

341. }

343. function IMT()
344. {
345. return  new Resource(DCTERM_NS . 'IMT');

347. }

349. function ISO3166()
350. {
351. return  new Resource(DCTERM_NS . 'ISO3166');

353. }

355. function ISO639_2()
356. {
357. return  new Resource(DCTERM_NS . 'ISO639-2');

359. }

361. function LCC()
362. {
363. return  new Resource(DCTERM_NS . 'LCC');

365. }

367. function LCSH()
368. {
369. return  new Resource(DCTERM_NS . 'LCSH');

371. }

373. function MESH()
374. {
375. return  new Resource(DCTERM_NS . 'MESH');

377. }

379. function PERIOD()
380. {
381. return  new Resource(DCTERM_NS . 'Period');

383. }

385. function POINT()
386. {
387. return  new Resource(DCTERM_NS . 'Point');

389. }

391. function RFC1766()
392. {
393. return  new Resource(DCTERM_NS . 'RFC1766');

395. }

397. function RFC3066()
398. {
399. return  new Resource(DCTERM_NS . 'RFC3066');

401. }

403. function TGN()
404. {
405. return  new Resource(DCTERM_NS . 'TGN');

407. }

409. function UDC()
410. {
411. return  new Resource(DCTERM_NS . 'UDC');

413. }

415. function URI()
416. {
417. return  new Resource(DCTERM_NS . 'URI');

419. }

421. function W3CDTF()
422. {
423. return  new Resource(DCTERM_NS . 'W3CDTF');

425. }


428. // DCMI Type Vocabulary

430. function COLLECTION()
431. {
432. return  new Resource(DCMITYPE_NS . 'Collection');

434. }

436. function DATASET()
437. {
438. return  new Resource(DCMITYPE_NS . 'Dataset');

440. }

442. function EVENT()
443. {
444. return  new Resource(DCMITYPE_NS . 'Event');

446. }

448. function IMAGE()
449. {
450. return  new Resource(DCMITYPE_NS . 'Image');

452. }

454. function INTERACTIVERESOURCE()
455. {
456. return  new Resource(DCMITYPE_NS . 'Interactive_Resource');

458. }

460. function MOVINGIMAGE()
461. {
462. return  new Resource(DCMITYPE_NS . 'Moving_Image');

464. }

466. function PHYSICALOBJECT()
467. {
468. return  new Resource(DCMITYPE_NS . 'Physical_Object');

470. }

472. function SERVICE()
473. {
474. return  new Resource(DCMITYPE_NS . 'Service');

476. }

478. function SOFTWARE()
479. {
480. return  new Resource(DCMITYPE_NS . 'Software');

482. }

484. function SOUND()
485. {
486. return  new Resource(DCMITYPE_NS . 'Sound');

488. }

490. function STILLIMAGE()
491. {
492. return  new Resource(DCMITYPE_NS . 'Still_Image');

494. }

496. function TEXT()
497. {
498. return  new Resource(DCMITYPE_NS . 'Text');
499. }

501. }


504. ?>
```

Documentation generated on Fri, 17 Dec 2004 16:14:44 +0100 by phpDocumentor 1.3.0RC3
